# Moderate, but Not Excessive, Training Attenuates Autophagy Machinery in Metabolic Tissues

**DOI:** 10.3390/ijms21228416

**Published:** 2020-11-10

**Authors:** Alisson L. da Rocha, Ana P. Pinto, Gustavo P. Morais, Bruno B. Marafon, Rafael L. Rovina, Allice S. C. Veras, Giovana R. Teixeira, José R. Pauli, Leandro P. de Moura, Dennys E. Cintra, Eduardo R. Ropelle, Donato A. Rivas, Adelino S. R. da Silva

**Affiliations:** 1 Postgraduate Program in Rehabilitation and Functional Performance, Ribeirão Preto Medical School, University of São Paulo (USP), Ribeirão Preto 14040-900, São Paulo, Brazil; alisson.rocha@usp.br (A.L.d.R.); anapp_5@usp.br (A.P.P.); gustavo.paroschi.morais@usp.br (G.P.M.); 2 School of Physical Education and Sport of Ribeirão Preto, University of São Paulo (USP), Ribeirão Preto 14040-900, São Paulo, Brazil; bruno.marafon@usp.br (B.B.M.); rafael.rovina@usp.br (R.L.R.); 3 Postgraduate Program in Movement Sciences, São Paulo State University (UNESP), Presidente Prudente 19060-900, São Paulo, Brazil; allicesaantos@hotmail.com (A.S.C.V.); giovana.rampazzo@unesp.br (G.R.T.); 4 Department of Physical Education, State University of São Paulo (UNESP), Presidente Prudente 19060-900, São Paulo, Brazil; 5 Laboratory of Molecular Biology of Exercise (LaBMEx), School of Applied Sciences, University of Campinas (UNICAMP), Limeira 13484-350, São Paulo, Brazil; jose.pauli@fca.unicamp.br (J.R.P.); mouralp@unicamp.br (L.P.d.M.); dennys.cintra@fca.unicamp.br (D.E.C.); eduardo.ropelle@fca.unicamp.br (E.R.R.); 6 Nutrition, Exercise, Physiology, and Sarcopenia Laboratory, United States, Tufts University, Boston, MA 02111, USA; donato.rivas@tufts.edu

**Keywords:** colchicine, exercise, concurrent training, overtraining, autophagy, endurance training, resistance training

## Abstract

The protective effects of chronic moderate exercise-mediated autophagy include the prevention and treatment of several diseases and the extension of lifespan. In addition, physical exercise may impair cellular structures, requiring the action of the autophagy mechanism for clearance and renovation of damaged cellular components. For the first time, we investigated the adaptations on basal autophagy flux in vivo in mice’s liver, heart, and skeletal muscle tissues submitted to four different chronic exercise models: endurance, resistance, concurrent, and overtraining. Measuring the autophagy flux in vivo is crucial to access the functionality of the autophagy pathway since changes in this pathway can occur in more than five steps. Moreover, the responses of metabolic, performance, and functional parameters, as well as genes and proteins related to the autophagy pathway, were addressed. In summary, the regular exercise models exhibited normal/enhanced adaptations with reduced autophagy-related proteins in all tissues. On the other hand, the overtrained group presented higher expression of *Sqstm1* and *Bnip3* with negative morphological and physical performance adaptations for the liver and heart, respectively. The groups showed different adaptions in autophagy flux in skeletal muscle, suggesting the activation or inhibition of basal autophagy may not always be related to improvement or impairment of performance.

## 1. Introduction

Moderate-intensity regular exercise is a well-known nonpharmacological strategy for increasing longevity, as well as treating and preventing a range of diseases [1,2]. Along with other stressful stimuli, physical exercise practice can also disturb the cellular homeostasis, which requires the autophagy mechanism for clearance and renovation of damaged cellular components, such as mitochondria and proteins [3]. The autophagy process is responsible for the turnover of organelles or portions of the cytosol, surrounded by a double membrane vesicle (i.e., autophagosome) that will fuse with lysosomes for material degradation [4]. Skeletal muscle autophagy is increased in response to exercise [5] and plays an essential role in the performance increment [6]. Indeed, other tissues are also impacted by exercise and exhibit the necessary adaptations to improve the entire metabolism [7]. Both the heart and liver have the autophagy pathway activated in response to exercise [8,9,10,11]. However, almost all available findings are related only to the endurance exercise model.

While autophagy maintains cellular energy homeostasis in response to acute exercise, its role in response to chronic exercise is related to the cycle of renewal of defective proteins and organelles, degrading them [12]. The autophagic response to physical activity in skeletal muscle presents a biphasic pattern and is marked by a rapid increase of the autophagic flux. This response occurs within minutes to hours and is mediated by post-translational modifications of the proteins responsible for this pathway [13]. Generally, after this initial stimulus, the activation of the transcriptional program of autophagic genes occurs in the long term to potentiate this autophagic response [5].

Continuous training is also essential to protect and treat some metabolic disorders such as high-fat diet-induced type 2 diabetes [14]. Interestingly, He and coworkers [11] showed that inducing autophagy is crucial to the benefits of exercise. These authors found that the positive effects of training on glucose tolerance did not occur in transgenic mice, unable to activate the autophagy pathway. Additionally, He et al. [15] showed that acute exercise is a powerful stimulus to induce autophagy, not only in skeletal muscle but also in the heart and liver. The evaluation of autophagy molecular markers without assessing autophagy flux directly has limitations since specific alterations on autophagic parameters may not reflect real changes in autophagic flux [16]. Although the effects of acute and chronic endurance exercise protocols on autophagic flux in vivo using lysosomal blockade have been addressed previously [17,18,19,20], the investigations regarding the impact of resistance, concurrent (resistance plus endurance), and exhaustive (overtraining) protocols on autophagic flux in vivo are unknown. Therefore, the main aim of the present investigation was to verify the chronic effects of endurance, resistance, concurrent, and overtraining protocols on basal autophagy flux in vivo in mice’s liver, heart, and skeletal muscle tissues. We hypothesized that the moderate exercise models (i.e., endurance, resistance, and concurrent) would improve the basal autophagy process since these protocols are generally related to positive adaptations of the body [7]. Regarding the chronic exhaustive exercise protocol, we expected impairment of the autophagy process in all tissues since this model is closely linked to disturbances on the whole metabolism [21].

## 2. Results

### 2.1. Except for Overtraining, the Other Protocols Increased Physical Performance

**Figure 1:**
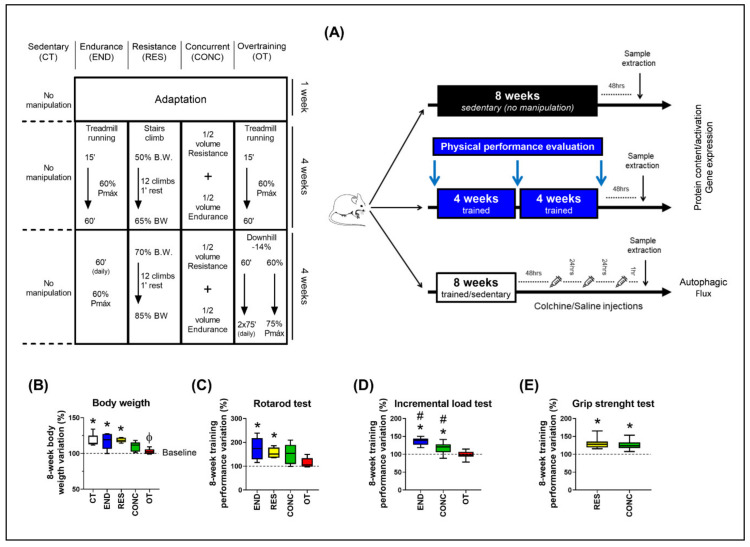
Schematic illustration of the experimental procedures (**A**). Body weight variation between week 8 and 0 (**B**). Performance variation (%) between weeks 8 and 0 for the incremental load test (**C**), rotarod test (**D**), and grip strength test (**E**). Data correspond to the mean ± SEM of 7–10 mice/group. * *p* ≤ 0.05 vs. baseline; Φ *p* ≤ 0.05 vs. CT and RES; # *p* ≤ 0.05 vs. other experimental groups. END (Endurance; submitted to the chronic endurance exercise protocol); RES (Resistance; submitted to the chronic resistance exercise protocol); CONC (Concurrent; submitted to the chronic concurrent exercise protocol); OT (Overtraining; submitted to the chronic exhaustive exercise protocol).

The body weight of the control (CT; sedentary), endurance (END; submitted to chronic endurance training), and resistance (RES; submitted to chronic resistance training) groups increased at week 8 compared to baseline. On the other hand, the body weight variation between weeks 8 and 0 for the overtraining (OT; submitted to chronic exhaustive training) group was lower compared to the CT and RES groups (Figure 1B). For the incremental load test, the END and concurrent (CONC; submitted to chronic concurrent training; i.e., a combination of endurance and resistance training) protocols increased the performance at week 8 compared to baseline. Additionally, the END and CONC groups presented higher values compared to the OT, while the END group was higher compared to the CONC group (Figure 1C). For the rotarod test, the END and RES protocols increased the performance at week 8 compared to baseline (Figure 1D). For the grip strength test, RES and CONC group increased the performance at week 8 compared to baseline (Figure 1E).

### 2.2. Resistance Training Protocol Induced Heart Functional Adaptations

The CONC protocol reduced the ejection fraction (%) at week 8 compared to weeks 0 and 4. The RES protocol increased the area (mm^2^) and volume at end-systole (µL) at week 8 compared to weeks 0 and 4. Additionally, this group increased the left ventricle mass (mg) at weeks 4 and 8 compared to week 0 and reduced fractional shortening at week 8 compared to week 0. The other parameters were not sensitive to the experimental protocols (Table S1).

### 2.3. Effects of Chronic Physical Exercise Protocols on Liver

**Figure 2:**
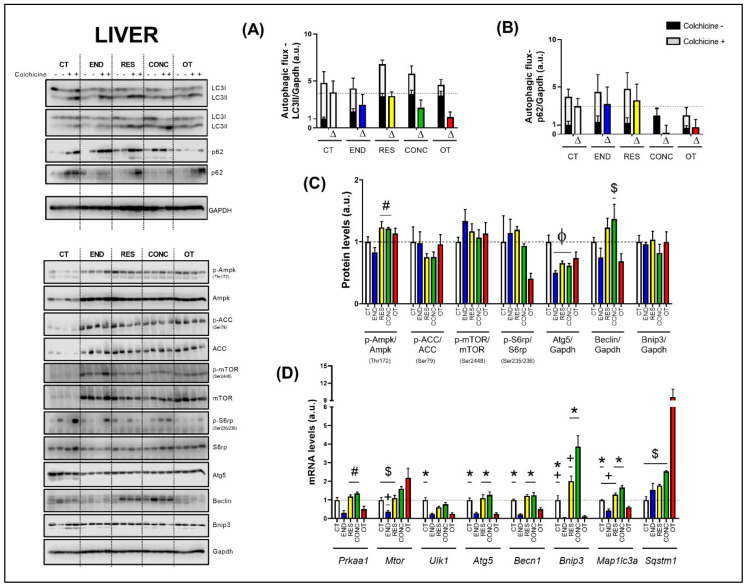
Autophagic flux for LC3II/Gapdh (**A**) and p62/Gapdh (**B**) in the liver. Protein levels of p-Ampk/Ampk, p-ACC/ACC, p-mTOR/mTOR, p-S6rp/S6rp, Atg5/Gapdh, Beclin/Gapdh, Bnip3/Gapdh in the liver (**C**). mRNA levels of *Prkaa1, Mtor, Ulk1, Atg5, Becn1, Bnip3, Map1lc3b*, and *Sqstm1* in the liver (**D**). Data correspond to mean ± SEM of 4 mice/group. For autophagic flux, there are animals treated with colchicine (colchicine +) and animals treated with saline (colchicine−). Δ represents the difference between colchicine (+) and saline (−). Φ *p* ≤ 0.05 vs. CT; # *p* ≤ 0.05 vs. END; * *p* ≤ 0.05 vs. END and OT; + *p* ≤ 0.05 vs. CONC; $ *p* ≤ 0.05 vs. OT. CT (Sedentary; control group); END (Endurance; submitted to the chronic endurance exercise protocol); RES (Resistance; submitted to the chronic resistance exercise protocol); CONC (Concurrent; submitted to the chronic concurrent exercise protocol); OT (Overtraining; submitted to the chronic exhaustive exercise protocol).

The basal autophagy flux in the liver was not modulated by the different chronic physical exercise protocols (Figure 2A,B). Regarding the protein levels (Figure 2C), the RES and CONC protocols increased the Ampk phosphorylation compared to the END protocol. The different chronic physical exercise protocols did not modulate the Acetyl-CoA Carboxylase (ACC), mTOR and ribosomal protein-S6 (S6rp) phosphorylation, and Bnip3 contents. The END, RES, and CONC protocols reduced the Atg5 contents compared to the CT protocol. The CONC group had higher Beclin content compared to the OT group.

Regarding the gene expressions (Figure 2D), the END protocol reduced the *Prkaa1* mRNA compared to the RES and CONC protocols. While the END group had lower *Mtor* mRNA compared to the CONC group, the OT group had higher *Mtor* mRNA compared to the CT, END, and RES groups. The END and OT protocols reduced the *Ulk1* mRNA compared to the CT protocol. The END and OT groups had lower *Atg5*, *Becn1*, *Bnip3*, and *Map1lc3a* mRNA than the other experimental groups. Additionally, the CT and RES protocols reduced the *Bnip3* mRNA compared to the CONC protocol. The CT and RES groups had lower *Map1lc3a* mRNA compared to the CONC group. The OT protocol increased the *Sqstm1* mRNA compared to the other experimental protocols.

**Figure 3:**
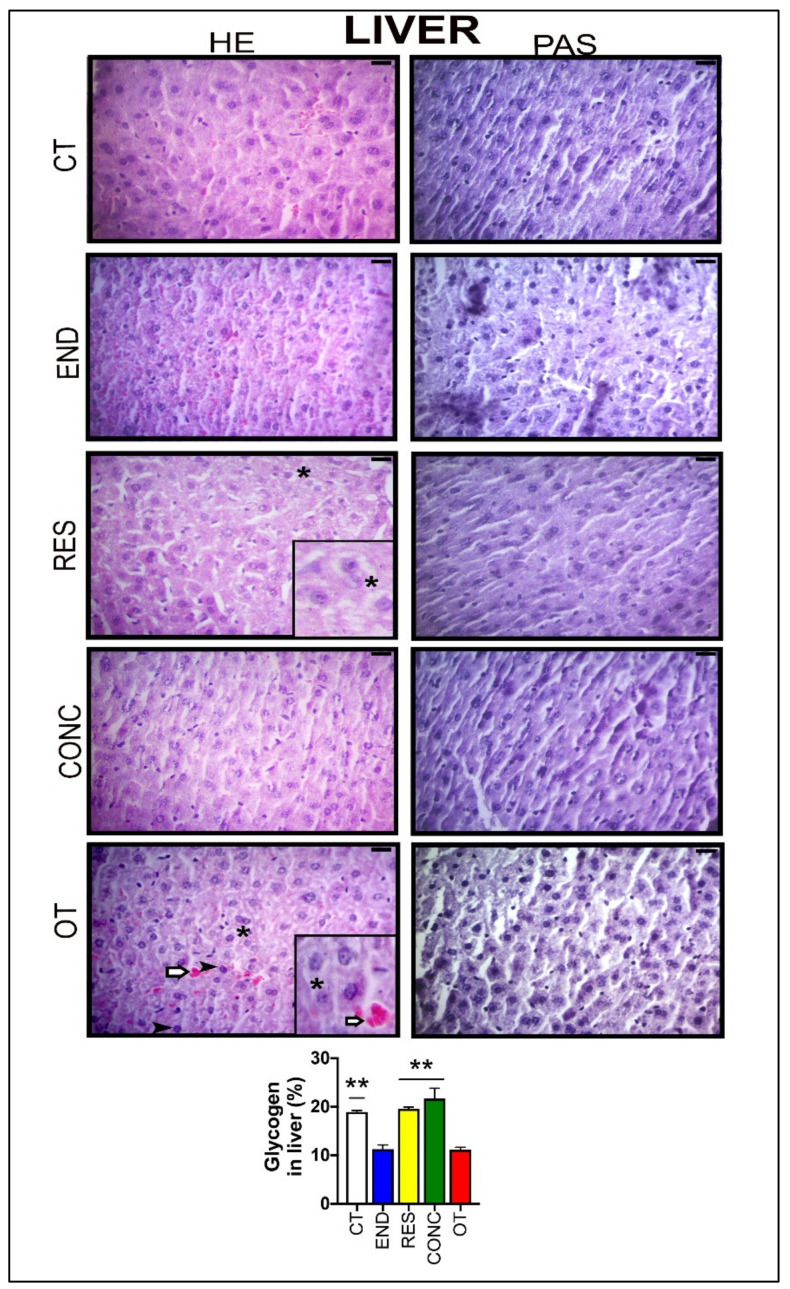
Histological characterization (×400) of the liver by hematoxylin-eosin (HE) and periodic acid Schiff (PAS) and, glycogen contents in the liver. Bar = 20µm. Data correspond to the mean ± SEM of 4–5 mice/group. Highlight 1.5× zoom. Cytoplasmic vacuoles (*). Hyperemia (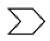). Nuclei with dense chromatin (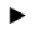) ** *p* ≤ 0.05 vs. END and OT; (Sedentary; control group); END (Endurance; submitted to the chronic endurance exercise protocol); RES (Resistance; submitted to the chronic resistance exercise protocol); CONC (Concurrent; submitted to the chronic concurrent exercise protocol); OT (Overtraining; submitted to the chronic exhaustive exercise protocol).

**Figure 4:**
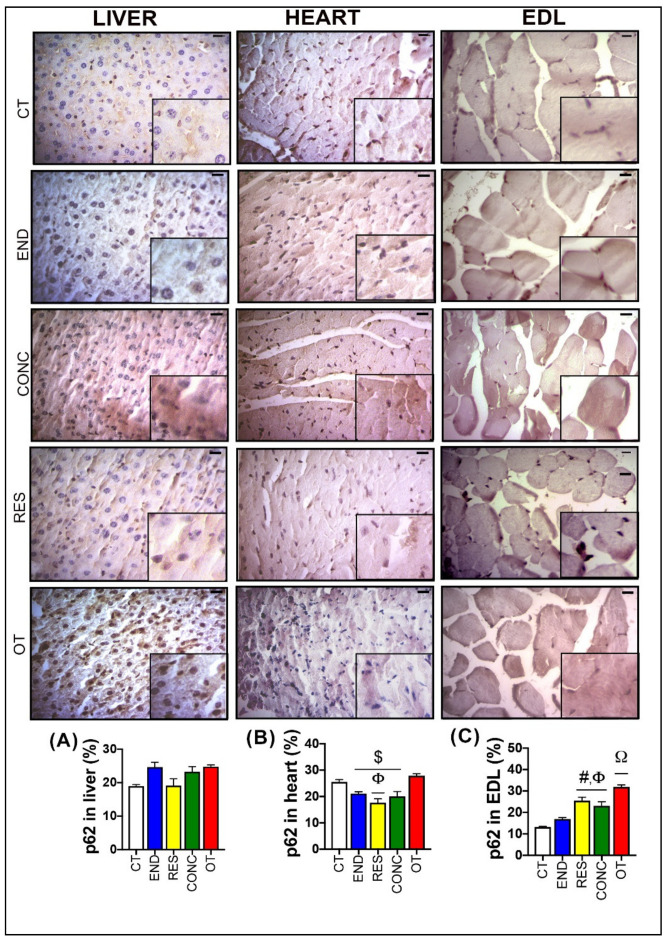
p62 expression by immunohistochemistry in the liver (**A**), heart (**B**), and EDL (**C**). Data correspond to mean ± SEM of 4–5 mice/group. Bar = 20 µm. Highlight 1.5× zoom. $ *p* ≤ 0.05 vs. OT. Φ *p* ≤ 0.05 vs. CT. # *p* ≤ 0.05 vs. END. Ω *p* ≤ 0.05 vs. all the other experimental groups. CT (Sedentary; control group); END (Endurance; submitted to the chronic endurance exercise protocol); RES (Resistance; submitted to the chronic resistance exercise protocol); CONC (Concurrent; submitted to the chronic concurrent exercise protocol); OT (Overtraining; submitted to the chronic exhaustive exercise protocol).

According to Figure 3, the hepatocytes of the CT group showed typical morphological features. The END and CONC groups displayed signs related to the presence of more Kupffer cells compared to the CT group. The RES group presented sings of hepatocytes with less cytoplasmic vacuoles (*) compared to the OT group. The OT group evidenced hepatocytes with cytoplasmic vacuoles (*), swelling and highest nuclei with dense chromatin (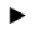), and hypertrophic hepatocytes. Additionally, the OT group presented sings of hyperemia for all analyzed samples, indicating a local increase in blood volume (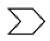). The CT, RES, and CONC groups showed higher glycogen percentages than the END and OT groups (Figure 3A). The p62 expression measured by the immunohistochemistry analysis was not different between the experimental groups (Figure 4A).

### 2.4. Effects of Chronic Physical Exercise Protocols on Heart

**Figure 5:**
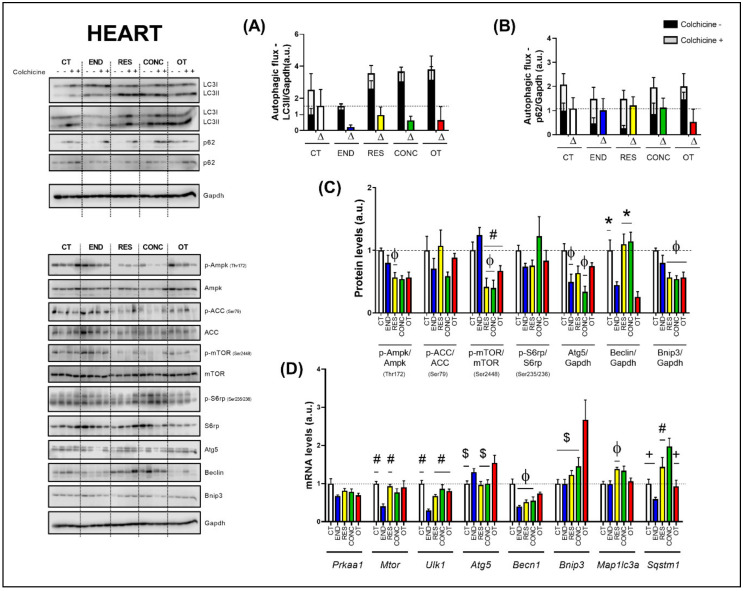
Autophagic flux for LC3II/Gapdh (**A**) and p62/Gapdh (**B**) in the heart. Protein levels of p-Ampk/Ampk, p-ACC/ACC, p-mTOR/mTOR, p-S6rp/S6rp, Atg5/Gapdh, Beclin/Gapdh, Bnip3/Gapdh in the heart (**C**). mRNA levels of *Prkaa1, Mtor, Ulk1, Atg5, Becn1, Bnip3, Map1lc3b*, and *Sqstm1* in the heart (**D**). Data correspond to the mean ± SEM of 4 mice/group. For autophagic flux, there are animals treated with colchicine (colchicine +) and animals treated with saline (colchicine −). Δ represents the difference between colchicine (+) and saline (−). Φ *p* ≤ 0.05 vs. CT; # *p* ≤ 0.05 vs. END; * *p* ≤ 0.05 vs. END and OT; + *p* ≤ 0.05 vs. CONC; $ *p* ≤ 0.05 vs. OT. CT (Sedentary; control group); END (Endurance; submitted to the chronic endurance exercise protocol); RES (Resistance; submitted to the chronic resistance exercise protocol); CONC (Concurrent; submitted to the chronic concurrent exercise protocol); OT (Overtraining; submitted to the chronic exhaustive exercise protocol).

The basal autophagy flux in the heart was not modulated by the different chronic physical exercise protocols (Figure 5A,B). Regarding the protein levels (Figure 5C), the END protocol decreased the Ampk phosphorylation compared to the CT protocol. The END group had higher mTOR phosphorylation compared to the RES, CONC, and OT groups. Additionally, the RES and CONC protocols reduced the mTOR phosphorylation compared to the CT protocol. The END and CONC groups had lower Atg5 contents compared to the CT group. The END and OT protocols reduced the Beclin contents compared to the other experimental protocols. The Bnip3 contents were lower for the RES, CONC, and OT groups compared to the CT group. The different chronic physical exercise protocols did not modulate the ACC and S6rp phosphorylation.

Regarding the gene expressions (Figure 5D), the *Prkaa1* mRNA was not modulated by the different chronic physical exercise protocols. The END protocol reduced the *Mtor* mRNA compared to the CT and RES protocols. The END group showed lower *Ulk1* mRNA compared to the other experimental groups. The OT protocol increased the *Atg5* mRNA compared to the CT, RES, and CONC protocols. The END, RES, and CONC groups had lower *Becn1* mRNA compared to the CT group. The OT protocol increased the *Bnip3* mRNA compared to the other experimental protocols. The RES group presented higher *Map1lc3a* mRNA compared to the CT group. While the END protocol reduced the *Sqstm1* mRNA compared to the other protocols, the CONC protocol increased the *Sqstm1* mRNA compared to the CT and OT protocols.

**Figure 6:**
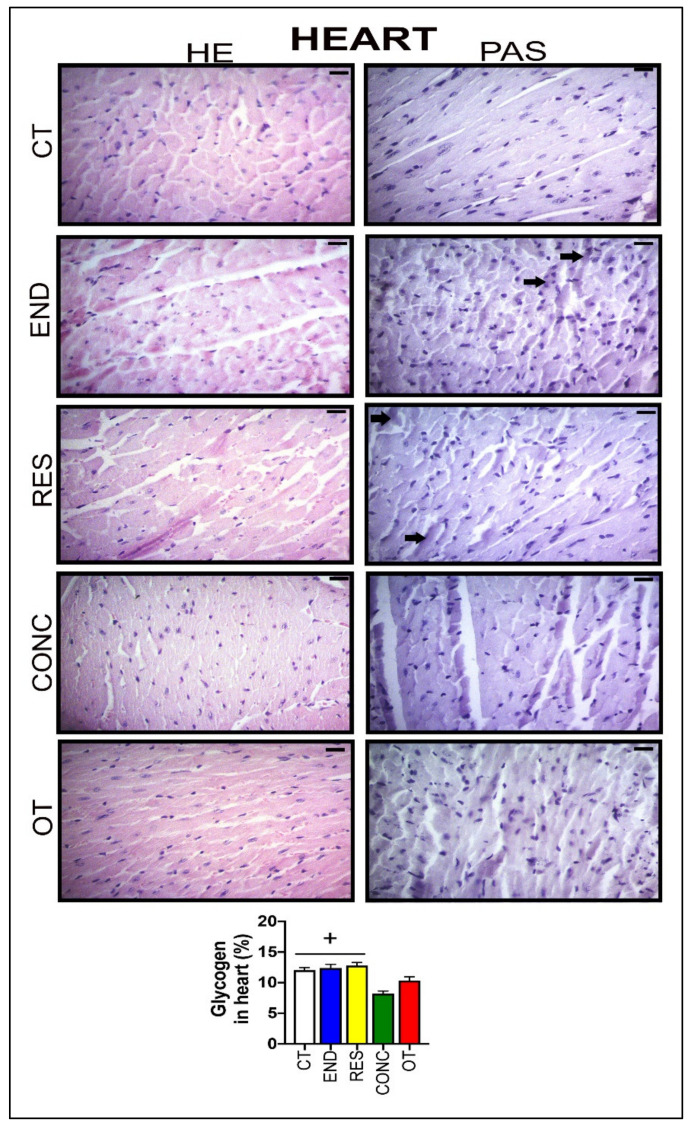
Histological characterization (×400) of heart by hematoxylin-eosin (HE) and periodic acid Schiff (PAS) and, glycogen contents in the heart. Bar = 20 µm. Data correspond to the mean ± SEM of 4–5 mice/group. Glycogen granules (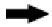). + *p* ≤ 0.05 vs. CONC. (Sedentary; control group); END (Endurance; submitted to the chronic endurance exercise protocol); RES (Resistance; submitted to the chronic resistance exercise protocol); CONC (Concurrent; submitted to the chronic concurrent exercise protocol); OT (Overtraining; submitted to the chronic exhaustive exercise protocol).

According to Figure 6, the heart samples of the CT group showed normal-looking fibers arranged in parallel and central nuclei. The END group displayed alterations related to the presence of cellular infiltration, which is indicative of tissue remodeling. The RES and CONC groups presented organized fibers with signs of hypertrophy. The OT group presented signs of polymorphonuclear cells, with lots of strung nuclei, and myocardial disarray. The END and RES groups showed higher amounts of glycogen granules extending from the intercalated discs (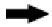) into the cells. The CT, END, and RES groups displayed a higher glycogen percentage than the CONC group (Figure 6). The p62 expression measured by the immunohistochemistry analysis was lower for the RES group compared to the CT group, but higher for the OT group compared to the END, RES, and CONC groups (Figure 4B).

### 2.5. Effects of Chronic Physical Exercise Protocols on EDL

**Figure 7:**
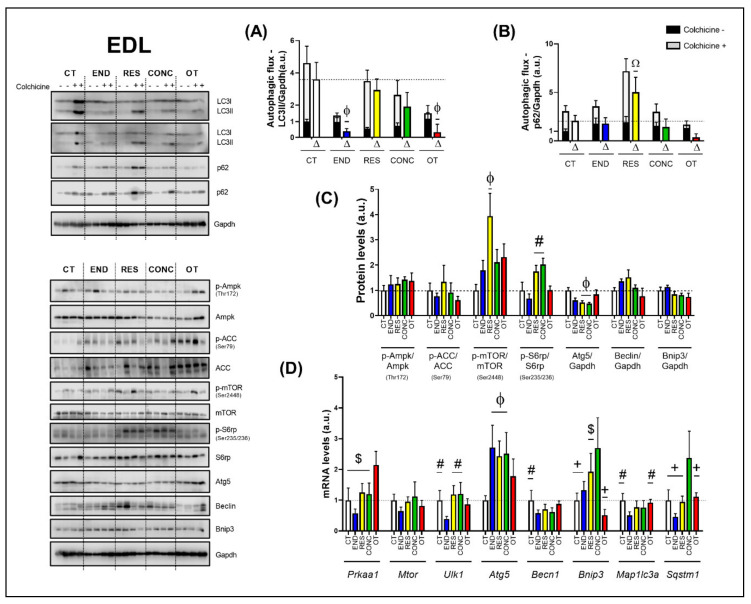
Autophagic flux for LC3II/Gapdh (**A**) and p62/Gapdh (**B**) in EDL. Protein levels of p-Ampk/Ampk, p-ACC/ACC, p-mTOR/mTOR, p-S6rp/S6rp, Atg5/Gapdh, Beclin/Gapdh, Bnip3/Gapdh in EDL (**C**). mRNA levels of *Prkaa1, Mtor, Ulk1, Atg5, Becn1, Bnip3, Map1lc3b*, and *Sqstm1* in EDL (**D**). Data correspond to the mean ± SEM of 4 mice/group. For autophagic flux, there are animals treated with colchicine (colchicine +) and animals treated with saline (colchicine −). Δ represents the difference between colchicine (+) and saline (−). Ω *p* ≤ 0.05 vs. other experimental groups; Φ *p* ≤ 0.05 vs. CT; # *p* ≤ 0.05 vs. END; + *p* ≤ 0.05 vs. CONC; $ *p* ≤ 0.05 vs. OT. CT (Sedentary; control group); END (Endurance; submitted to the chronic endurance exercise protocol); RES (Resistance; submitted to the chronic resistance exercise protocol); CONC (Concurrent; submitted to the chronic concurrent exercise protocol); OT (Overtraining; submitted to the chronic exhaustive exercise protocol).

The END and OT protocols reduced the autophagic flux for LC3II compared to the CT group (Figure 7A). Additionally, the RES group increased the autophagic flux for p62 compared to the other experimental groups (Figure 7B). Regarding the protein levels (Figure 7C), the Ampk phosphorylation, Beclin, and Bnip3 contents were not modulated by the different chronic physical exercise protocols. While the RES protocol increased the mTOR phosphorylation compared to the CT protocol, the RES and CONC protocols increased the S6rp phosphorylation when compared to the END group, and both groups reduced the Atg5 contents compared to the CT protocol.

Regarding the gene expressions (Figure 7D), the OT protocol increased the *Prkaa1* mRNA compared to the other experimental protocols. The different chronic physical exercise protocols did not modulate the *Mtor* mRNA. The END protocol decreased the *Ulk1* mRNA compared to the CT, RES, and CONC protocols. The END, RES, and CONC groups had higher *Atg5* mRNA compared to the CT group. The END protocol reduced the *Becn1* mRNA compared to the CT protocol. The CONC group had higher *Bnip3* mRNA compared to CT, END, and OT groups. Additionally, the OT group had lower *Bnip3* mRNA compared to the RES group. The END protocol reduced the *Map1lc3a* mRNA compared to the CT and OT protocols. The CONC group had higher *Sqstm1* mRNA compared to the other experimental groups.

**Figure 8:**
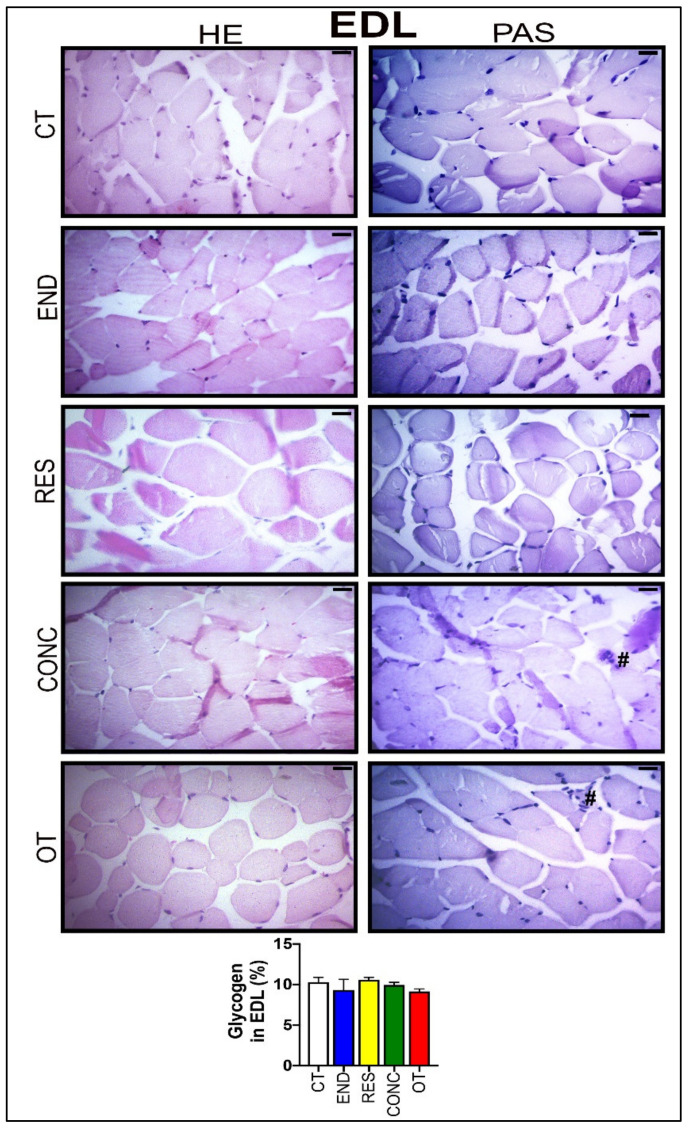
Histological characterization (×400) of EDL by hematoxylin-eosin (HE) and periodic acid Schiff (PAS), and glycogen contents in the EDL. Bar = 20 µm. Data correspond to the mean ± SEM of 4–5 mice/group. Polymorphonuclear cells (#). (Sedentary; control group); END (Endurance; submitted to the chronic endurance exercise protocol); RES (Resistance; submitted to the chronic resistance exercise protocol); CONC (Concurrent; submitted to the chronic concurrent exercise protocol); OT (Overtraining; submitted to the chronic exhaustive exercise protocol).

According to Figure 8, the EDL samples of the CT group showed polygonal fiber aspects, with peripheral nuclei, surrounded by endomysium. The END group displayed polygonal fiber aspects, infiltrated inside the fibers characterizing the normal regeneration process. The RES group presented rounded fibers, possibly due to increased protein synthesis of the muscle fibers. The CONC group presented a decrease in the connective tissue (endomysium and perimysium), indicating a possible increase in protein synthesis. The OT group showed rounded fibers with polymorphonuclear cells (#) and increased the diameter of muscle fibers in a cross-section of the muscle. The groups did not present significant differences in the glycogen contents (Figure 8). The p62 expression measured by the immunohistochemistry analysis was higher for the RES and CONC groups compared to the CT and END group. Additionally, the OT group had higher p62 expression compared to the other experimental groups (Figure 4C).

## 3. Discussion

Moderate-intensity regular exercise-induced autophagy is considered a potent nonpharmacological intervention strategy to counteract several pathologies and enhance lifespan [1,2]. Once autophagic regulation can occur in one or more of its five steps (i.e., initiation, elongation, maturation, fusion, and degradation), the evaluation of autophagy flux in vivo is fundamental for a deep understanding of this lysosomal degradation signaling pathway in different experimental approaches [16,22]. Therefore, we investigated the responses of basal autophagy flux in vivo to endurance, resistance, concurrent, and excessive training in metabolic tissues of rodents. Taken together, these findings demonstrate that the type of chronic physical exercise protocol plays a vital role in adapting the basal autophagy flux in vivo in mice’s skeletal muscles.

The CT, END, and RES groups increased their body weights from week 0 to week 8. On the other hand, the OT model led to a lower gain of body weight in the same period. As previously investigated by Pereira and coworkers [23], this latter result may be related to chronic excessive exercise-induced hypothalamic inflammation. According to the improvement of incremental load, rotarod, and grip strength tests, the END, RES, and CONC protocols were efficient. As previously published [24], the OT model impaired the enhancement of the incremental load test performance.

### 3.1. Effects of Chronic Physical Exercise Protocols on Liver

Hepatic autophagy activation protects hepatocytes from injury and cell death, as well as reducing lipid accumulation and fibrosis in nonalcoholic fatty liver disease [25]. Previous investigations have described the positive effects of endurance exercise on liver fat reduction by activating AMPK and autophagy pathway in diet-induced obese mice [26,27]. Other authors have verified hepatic autophagy induction after endurance exercise in mice fed with a standard chow diet [8,9]. However, these studies focused only on the acute responses of the endurance exercise in autophagy machinery.

Our study showed that mice that participated in the endurance and overtraining protocols reduced most of the mRNA expressions related to several steps of the autophagy process. Additionally, these models decreased glycogen concentrations. Altogether, these changes may be related to the higher training volume performed by the END and OT groups. In contrast to the concept that mRNA expression alterations range from 3 to 12 h after exercise stimulus cessation [13], we observed several responses even after a 48-h rest period. These data support the theory that chronic exercise based on endurance stimulus is an epigenetic factor that downregulates the fundamental autophagic genes expression in the liver to new baseline status.

The mRNA expression reductions for these groups can be an adaptative response to exercise, since these alterations did not lead to significant changes in autophagic flux. Furthermore, the three moderate/regular exercise models (i.e., endurance, resistance, and concurrent) also reduced the Atg5 protein content, suggesting an economical way to maintain the functioning of this lysosomal degradation signaling pathway with reduced energy demand for the processes of gene transcription and protein translation. Conversely, even with the downregulation of several autophagic genes, the OT group showed an upregulation around 2-fold for *Mtor* and 12-fold for *Sqstm1*, which can be detrimental to the liver function. The increased expression of *Mtor* supports our previous data showing the chronic exhaustive exercise activating Akt/mTOR signaling pathway and leading to hepatic fat accumulation [28]. In the present study, we did not observe differences in hepatic mTOR activation, but this could be due to the different liver extraction times (36 h vs. 48 h) after the grip force test.

The *Sqstm1* codifies the p62 protein, which is responsible for recognizing cellular waste and sequestrating them to lysosomal degradation through the autophagy process [29]. Komatsu et al. [30] elegantly showed that p62 accumulation could be detrimental in mouse livers, leading to a pathological phenotype and playing a role in oxidative stress response, causing hepatocytes damage [29]. Here, we did not show increased p62 accumulation in the cell, but the abnormal upregulation of *Sqstm1* expression could lead to a detrimental status in a more extended period. Future studies should evaluate the effects of constant activation of transcriptional machinery of p62 to elucidate this outcome.

Histology and immunohistochemical were performed to evaluate morphological, morphometric, and cellular alterations in the tissues of the experimental models. Komine and coworkers [31] previously described the beneficial effects of endurance exercise on Kupffer cells. In addition to the END protocol, we verified the CONC model increased the presence of these cells, which are responsible for the initial steps of the innate immune response. In addition to the preceding findings [28], here, the OT protocol led to negative adaptations such as hepatocytes with cytoplasmic vacuoles, swelling, and highest nuclei with dense chromatin, as well as hyperemia.

### 3.2. Effects of Chronic Physical Exercise Protocols on the Heart

Basal autophagy plays a vital role in cardiac myocyte function and survival. For instance, the ablation of autophagy-related genes is linked to pathological cardiac hypertrophy and dysfunction in rodents [32]. In response to stress, autophagy activation is necessary to adapt to an increase in nutritional and energy demands via protein catabolism [3]. Yan et al. [33] verified that autophagy has a vital function in endurance exercise-induced cardiac protection, regulating cardiac fibrosis, fetal gene reprogramming, and mitochondrial biogenesis. Furthermore, Li and coworkers [10] concluded that the basal autophagy machinery in cardiac muscle was improved in chronic exercise based on high-intensity interval training but not in continuous moderate-intensity training, evidencing that the adaptations may be dependent on the exercise intensity.

In the present study, we showed that the autophagy-related proteins for all exercise training models were not increased compared to the CT group. These alterations corroborate our findings in the liver, suggesting an improvement in the autophagy process. The cell quality control was maintained for the END, RES, and CONC groups with a reduction of one or more autophagy-related proteins, since the experimental groups did not present alterations in autophagic flux, as well as showed normal or enhanced morphological adaptations and cardiac function. In addition, the END group showed lower baseline values for several autophagy-related genes analyzed.

On the other hand, even with similar adaptations in protein content, the OT protocol led to polymorphonuclear cells with lots of strung nuclei and myocardial disarray, reinforcing the negative morphological adaptation to chronic excessive exercise [34]. Li et al. [35] verified that an acute exhaustive exercise session suppressed autophagy, which was linked to ischemia-hypoxia injury in rat myocardium. In contrast, exhaustive overload-exercise-induced autophagy was related to disrupted cardiomyocytes [36]. According to Sciarretta and coworkers [37], the functional implications of the induction or suppression of the autophagy pathway in the heart under different experimental conditions must be further investigated.

Interestingly, the OT protocol increased around 3-fold the *Bnip3* gene expression but reduced the Bnip3 protein levels. This protein is responsible for inducing cell death and mitochondrial dysfunction [38]. However, Hanna et al. [39] evidenced an exciting role in which this protein protects cell death through mitophagy induction. The Bnip3 localized at mitochondria acts as an autophagy receptor, binding to LC3 on the autophagosome and favoring the autophagy process, as well as mitochondria degradation on autophagolysosomal breakdown [39]. Here, we hypothesize some molecular mechanisms of translational control impair Bnip3 formation even with high levels of *Bnip3* mRNA. With a compromised production of Bnip3, the stress induced by exhaustive training can lead to an increased content of dysfunctional mitochondria producing a harmful ambient in cell and, ultimately, leading to the negative previously described morphological adaptations in cardiomyocytes.

The autophagy flux evaluation by using lysosome suppressors is crucial for a profound interpretation of the autophagy pathway adaptations to different stimuli [16,22,37]. Ferreira and coworkers [20] verified that a chronic endurance exercise model in infarcted mice could reestablish cardiac autophagic flux [20]. To the best of our knowledge, this is the first time that the cardiac autophagic flux using a lysosome inhibitor is assessed in healthy mice submitted to different training models.

### 3.3. Effects of Chronic Physical Exercise Protocols on EDL

It is well known that autophagy plays an essential role in physical exercise-mediated skeletal muscle positive adaptations [6]. Interestingly, the END group exhibited the same pattern observed in the other metabolic tissues, reducing several autophagy-related genes in skeletal muscle. In addition, this group showed reduced autophagic flux with lower LC3-II content. However, all groups submitted to moderate training presented higher *Atg5* mRNA expression. Corroborating the current data, Kim and coworkers [17] observed that seven days of chronic contractile activity decreased the tibialis anterior muscle autophagy flux, and justified that the exercise-mediated metabolic signaling pathway attenuation is a characteristic of adapted muscle [40]. In contrast, Ju et al. [19] verified that eight weeks of swimming training increased triceps muscle autophagy flux. These diverging findings may be associated with the use of different exercise models (i.e., running, chronic contractile activity, or swimming), skeletal muscle types (i.e., EDL, tibialis anterior, or triceps), and sample extraction times after exercise cessation (i.e., 48 h, 90 min, or 48 h).

The RES group had higher activation of mTOR in this tissue. Both RES and CONC groups exhibited higher S6rp phosphorylation (an mTOR signaling pathway target), suggesting an increase of protein synthesis. Even without a direct method to measure this adaptation, the morphological analyses support this finding presenting evidence of muscle hypertrophy for both groups. Furthermore, the RES group also showed increased p62 immunoexpression and its accumulation at basal levels, when induced by colchicine treatment. Classically, the p62 is a protein that signals, in the early stages of the autophagy process, the cargo that must be degraded by binding directly to LC3-I and forming the autophagosome [41]. However, Duran et al. [42] elegantly showed that p62 also acts as a signaling adaptor activating the mTOR signaling pathway. It is an integral part of the process of starting this pathway mediating amino acid signaling. Our findings regarding p62 content support mTOR activation in EDL, leading to protein synthesis and signs of skeletal muscle hypertrophy. The CONC group also showed higher p62 immunoexpression and signs of increased protein synthesis, but with concomitant upregulation of some autophagy-related genes. Recently, Ranjbar et al. [43] did not find significant changes for the mRNA levels and protein contents of LC3BI, LC3BII, and p62 in gastrocnemius and tibialis muscles of healthy rodents performing six weeks of concurrent training. Indeed, this is the first time that the responses of autophagic flux in vivo to resistance and concurrent exercise are explored.

Previously, this OT model led to atrophy signs, endoplasmic reticulum stress, hypertrophy inhibition, inflammation, and insulin signaling impairment in EDL samples [24,44,45,46]. Here, this same skeletal muscle type presented an increase in *Prkaa1* mRNA level and p62 immunoexpression, as well as a reduction in autophagy flux. Although both END and OT protocols reduced the autophagy flux in vivo, the first increased physical performance and the latter impaired it. Furthermore, the OT group showed an exciting relationship between autophagy flux reduction and concomitant p62 content increase (demonstrated by IHC experiments), suggesting an interruption/impairment of the autophagy process. Zhang and coworkers [47] verified that one week of exhaustive exercise increased the mRNA levels of atrophy markers and protein contents of Beclin, Bnip3, and LC3-II. Taken together, these findings suggest that the activation or inhibition of basal autophagy in response to exercise may not always be related to improvement or impairment of performance.

## 4. Materials and Methods

### 4.1. Experimental Animals

Eight-week-old male C57BL/6 mice from the Central Animal Facility of the Ribeirão Preto campus from the University of São Paulo (USP) were accommodated in sterile micro-insulators (three animals per cage) in a ventilated rack (INSIGHT^®^, Ribeirão Preto, São Paulo, Brazil) with controlled temperature (22 ± 2 °C) on a 12:12-h light-dark normal cycle (light: 6 a.m. to 6 p.m., dark: 6 p.m. to 6 a.m.). Food (Purina chow) and water were provided ad libitum. All experimental procedures were performed respecting the Brazilian College of Animal Experimentation (COBEA) and were approved by the Ethics Committee of the University of São Paulo (I.D 2017.5.30.90.8). Mice were divided into five experimental groups: Sedentary (CT; Control group), Endurance (END; submitted to chronic endurance training), Resistance (RES; submitted to chronic resistance training), Concurrent (CONC; submitted to chronic concurrent training; i.e., a combination of endurance and resistance training) and Overtraining (OT; submitted to chronic exhaustive training). The schematic illustration of the experimental procedures is presented in Figure 1A.

### 4.2. Physical Performance Evaluation

Mice from the END, CONC, and OT groups were adapted to a 1-week period on a treadmill (INSIGHT^TM^, Ribeirão Preto, SP, Brazil) for five days, 10 min/day, at a speed of 6 m.min^−1^. Additionally, mice from the RES group were submitted to a 1-week adaptation period on a ladder-climbing (INSIGHT^TM^, Ribeirão Preto, SP, Brazil), 6 times/day without an external load attached to the animal’s tail. The ladder had an 80° inclination and 85 steps with a distance of 6 mm between each. The control group did not undergo any experimental manipulation to ensure no interference from the acute bout of physical tests on the molecular parameters since these are sedentary mice. The efficiency or not of each exercise protocol in increasing the performance was evaluated comparing their basal condition (i.e., the results of the physical tests before any experimental manipulation) to their results after each chronic exercise protocol.

#### 4.2.1. Incremental Load Test

The incremental load test was performed before (week 0), in the middle (week 4), and at the end (week 8) of the training protocols for END, CONC, and OT groups. Forty-eight hours after the last training/adaptation session, the incremental load test started at an initial velocity of 6 m/min with increments of 3 m/min every 3 min until voluntary exhaustion. The END and CONC groups performed the test at 0% of inclination in all evaluations, while the OT group performed at 0% at week 0 and −14% of inclination at weeks 4 and 8. The exhaustion velocity was used to prescribe the exercise intensities for the END, CONC, and OT groups. RES group did not perform this physical performance test because the incremental load test evaluates the aerobic power, which is not the physical capacity focused on resistance-based training programs.

#### 4.2.2. Rotarod Test

Twenty-four hours after the incremental load test, the motor coordination and balance were evaluated using an accelerating single-station rotarod treadmill (INSIGHT^®^, Ribeirão Preto, São Paulo, Brazil). Mice were placed on the rotarod apparatus with an initial velocity of 1 rpm and a final velocity of 40 rpm, which was achieved 300 s after the rotarod test beginning. Mice performed three trials, and the average time that each rodent was able to stay on the top of the rotarod apparatus was recorded [23].

#### 4.2.3. Grip Strength Test

Four hours after the rotarod test, the experimenter gently held mice by the tail and allowed them to grasp the horizontally positioned metal bar of the Grip Strength System (Avs Projetos^®^, São Carlos, São Paulo, Brazil) with the four paws. Each mouse performed three trials for adaptation and three trials for force measurement with 3–5 min of recovery between each attempt. The highest force value applied to the metal bar was registered as the peak tension (N) and was used as a performance parameter [45]. END and OT group did not perform this physical performance test because the grip strength test evaluates the strength of the mice, which is not the physical capacity focused on endurance-based training programs.

### 4.3. Chronic Physical Exercise Protocols

#### 4.3.1. Endurance Training Protocol

Each experimental week consisted of five days of training, followed by two days of recovery. Mice from the END group ran at 60% of exhaustion velocity (EV) at 0% of inclination for 15 min in the first week, 30 min in the second week, 45 min in the third week, and 60 min from the fourth to eighth week [48].

#### 4.3.2. Resistance Training Protocol

Each experimental week consisted of five days of training, followed by two days of recovery. The protocol started with one stair climb without load (warmup) followed by 12 stairs climbs with 1–2 min of recovery between each set. The external load was attached to the mice’s tail and consisted of 50% of the body weight in the first week, followed by increments of 5% of their body weight per week. This protocol was adapted from Khamoui et al. [49].

#### 4.3.3. Concurrent Training Protocol

Each experimental week consisted of five days of training, followed by two days of recovery. The concurrent protocol consisted of half the volume of the resistance exercise protocol followed immediately by half the volume of the endurance exercise protocol. Thus, mice performed one stair climb without load to warmup followed by 6 stairs climbs with 50% of the body weight at week 1, which increased by 5% of body weight in each experimental week until the end of the protocol. After stair climbing, mice ran at 60% of EV for 7.5 min and a half at week one, increasing 7 min and a half per week until week 4. The animals ran 30 min at 60% of EV from week 4 to week 8.

#### 4.3.4. Overtraining Protocol

Each experimental week consisted of five days of training, followed by two days of recovery. The overtraining protocol lasted eight weeks and was performed as previously described [24]. Briefly, mice started the protocol running for 15 min at 60% of EV at 0% of inclination at week 1. Progressively until week 4, the mice had increments of 15 min per week. From week 5 to 8, mice increased up to 75% of EV with two daily sessions of 75 min at −14% of inclination.

### 4.4. Echocardiogram

Mice from all experimental groups did the exam before (week 0), in the middle (week 4), and at the end of the exercise training protocols (week 8). The exam was performed using a Vevo 2100^®^ ultrasound system (VisualSonics, Toronto, ON, USA) with a 30 Mhz transducer on a heated platform with monitoring of vital signs. The animals were anesthetized with isoflurane inhalation anesthetic at 3% of the chamber volume, and the loss of the foot reflex was the parameter to evaluate the efficacy of the anesthesia. After being anesthetized, the animals were fixed in the supine position on the platform and trichotomized. During the procedure, the animals inhaled isoflurane enriched with a flow of 5 L/min of oxygen. Two-dimensional (B-Mode) and M-mode (M-Mode) images were acquired on the parasternal long axis using the center of the left ventricle (LV) as reference. The variables measured in the M-mode were: LV mass, left ventricular internal dimension at the end of the diastole and systole, LV ejection fraction, interventricular septum thickness at the end of the diastole and systole, fractional shortening, and left ventricular posterior wall thickness at the end of the diastole and systole. In the B-Mode, the analyzed variables were: heart rate, cardiac output, systolic and diastolic volume, and stroke volume.

### 4.5. Liver, Heart, and Skeletal Muscle Extraction

Forty-eight hours after the grip strength test, the animals were anesthetized by an intraperitoneal administration of xylazine (10 mg/kg body weight) and ketamine (100 mg/kg body weight). As soon as the loss of pedal reflexes confirmed the effect of anesthesia, the liver, heart (with subsequently left ventricle isolation), and extensor digitorum longus (EDL) were harvested, washed with saline, and specifically stored for reverse transcription-quantitative polymerase chain reaction (RTq-PCR), immunoblotting, immunohistochemistry and histology analysis.

### 4.6. Reverse Transcription–Quantitative Polymerase Chain Reaction (RT-qPCR)

Total RNA was extracted with Trizol (Invitrogen, Carlsbad, CA, USA), and the cDNA was synthesized with 1000 ng of total RNA using the High-Capacity cDNA Reverse Transcription Kit (Applied Biosystems, Foster City, CA, USA) according to the manufacturer instructions. Quantitative real-time PCR was performed on the StepOnePlusTM Real-Time PCR System (Applied Biosystems, Foster City, CA, USA) to analyze the relative mRNA expression of the following genes: *Prkaa1* (protein kinase, AMP-activated, alpha 1 catalytic subunit), *Mtor* (mechanistic target of rapamycin kinase), *Ulk1* (unc-51 like kinase 1), *Atg5* (autophagy-related gene 5), *Becn1* (Beclin1), *Bnip3* (BCL2 interacting protein 3), *Map1lc3b* (microtubule-associated protein 1 light chain 3 beta), and *Sqstm1* (sequestosome 1/p62) (Table S2).

The 5× HOT FIREPol EvaGreen qPCR SuperMix from Solis BioDyne (Tartu, Estonia) reagent was used following the manufacturer’s specifications for these reactions. The cycles for these PCRs were as follows: one cycle at 12 °C for 12 min, 40 cycles of 15 s at 95 °C, 25 s at 60 °C and 25 s at 72 °C. A melting curve was performed right after. *Gapdh* (glyceraldehyde-3-phosphate dehydrogenase) was used as a reference gene for the normalization of the data. Each PCR assay was performed in duplicate. Relative quantification was calculated by the 2 ^−ΔΔCT^ method using the Thermo Fisher Cloud Software, RQ version 3.7 (Life Technologies Corporation, Carlsbad, CA, USA).

### 4.7. Immunoblotting

The immunoblotting technique was performed as previously described [23,34]. The primary antibodies used were: p-Ampk (Thr172; #2531), Ampk (SC-74761), p-ACC (Ser79; #3661), ACC (#3662), p-mTOR (#2971), mTOR (#2972), p-S6rp (ser235/236; SC-293144), S6rp (SC-74459), Atg5 (#12994), Beclin (66665-1-1g), Bnip3 (ab38621), Lc3b (#2775), SQSTM1/p62 (#23214), and Gapdh (#2118). The secondary antibodies used were: Anti-rabbit IgG (#7074) and Anti-mouse IgG (#7076). The antibodies were from Cell Signaling Technology (Cell Signaling Technology, MA, USA), Abcam (Abcam, Cambridge, UK) or Proteintech (Proteintech Group, IL, USA). All the primary antibodies were used at a dilution of 1:1000, and the secondary antibodies were used at a dilution between 1:5000 to 1:2000. Images were acquired by the C-Digit Blot Scanner (LI-COR, Lincoln, NE, USA) and quantified using the software Image Studio for C-DiGitTM Blot Scanner.

### 4.8. Histological Analyses

Samples of liver, heart (left ventricle), and EDL were fixed in 10% formaldehyde dissolved in phosphate buffer saline (0.1 M pH 7.3) for 24 h. The samples were dehydrated in a graded ethanol series, cleared in xylene, and embedded in Paraplast (Sigma Co, Saint Louis, MO, USA). For histopathological evaluation, 5-μm slides were stained with hematoxylin and eosin (HE) and periodic acid-Schiff (PAS) for glycogen analysis. The analyses were performed using a photomicroscope digital Axiophot II Zeiss Microscope (One Zeiss Drive, Thornwood, NY, USA). Images were acquired at 40x resolution, the intensity of staining of glycogen was examined in 10 fields per animal using Image-J software version 1.50i (National Institutes of Health, Bethesda, MD, USA), and the color threshold was set to identify glycogen (i.e., magenta stain from PAS), which was converted to a black/white image and set to binary for measurement. The binary image was compared to the original, confirming both the scope and intensity of glycogen staining. Glycogen was reported as a percentage of the area marked by staining in the binary image, which could then be compared to the total area of the image.

### 4.9. Immunohistochemistry

Antigenic recovery was performed in a microwave (15 min) or pressure cooker for all the tissues. Endogenous peroxidase activity was blocked with 3% of hydrogen peroxide diluted in methanol for 15 min. Subsequently, the tissues were blocked with 3% goat serum, diluted in TBS-T (1% Triton X-100, 100 mM of Tris, pH 7.4) for 1 h and incubated with overnight a polyclonal primary antibody anti-rabbit SQSTM1/p62 (1:200 in heart and 1:100 in liver and EDL; Cell Signaling Technology, MA, USA) diluted in BSA 1% diluted with TBS-T. The following day, the sections were incubated with a secondary goat anti-rabbit HRP antibody (Santa Cruz sc-2030; 1:200) diluted in BSA 1% for two hours.

The sections of each experimental group were evaluated through the brownish precipitate of diaminobenzidine that was used as the chromogen indicating immunoreactivity and then stained with hematoxylin. For all markers, positive and negative controls were performed. Color selection was used to select and reserve the positive color pixels while the background color pixels were eliminated. The statistical color model was created based on the histogram of these reserved positive color pixels. The result was in % staining intensity for the image. The samples were acquired using a photomicroscope digital Axiophot II Zeiss Microscope (One Zeiss Drive, Thornwood, NY, USA) at 40x resolution, the intensity of immunoreactivity of SQSTM1/p62 antigen was examined in 10 fields per animal using Image-J software version 1.50i (National Institutes of Health, Bethesda, MD, USA), and the percentage of tissue marking was quantified for each image.

### 4.10. Autophagic Flux

Forty-eight hours after the last exercise session of each training protocol, mice were treated with intraperitoneal injections of colchicine (0.4 mg/kg/day; AB120663, Abcam, Cambridge, UK) or vehicle (0.9% saline) for 3 consecutive days. One hour after the last injection of colchicine, the rodents were anesthetized as previously described, and the liver, heart (with subsequently left ventricle isolation), and EDL were harvested, washed with saline, and used for the measurement of the protein levels of LC3-II (the active/lipidated form of LC3-I) and p62 (autophagosome adaptor protein) by the immunoblotting technique. The schematic presentation of colchicine treatment is shown in Figure 1B. In addition, there were specific mice used in this analysis, and they did not perform physical tests. The colchicine values were subtracted from the vehicle mean values of corresponding protocols (for example, mean END with the colchicine–mean END with the saline) for autophagic flux calculation.

### 4.11. Statistical Analysis

Results are expressed as the mean ± standard error of the mean (SEM). The Shapiro-Wilk’s W-test was used to verify data normality, and Levene’s test was used to test the homogeneity of variances. When normality was confirmed, one-way, or repeated-measures analysis of variance (ANOVA) was used to examine the differences between the experimental groups or time points. All statistical analyses were two-sided, and the significance level was set at *p* ≤ 0.05. Statistical analyses were performed using the software GraphPad Prism for Windows (GraphPad Software, version 8.0.1, GraphPad Software, San Diego, CA, USA).

## 5. Conclusions

**Figure 9:**
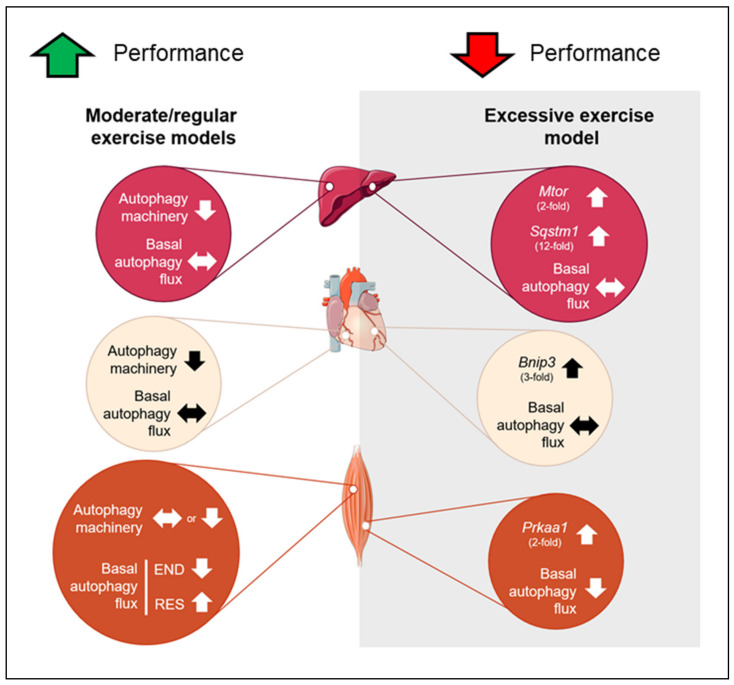
Schematic presentation of the main findings. Red circles represent the adaptations in liver, cream circles represent the adaptations in heart and orange circles represent the adaptations in skeletal muscle for moderate and excessive exercise models. Up arrows indicate upregulation, down arrows indicate downregulation and side arrow indicate no alteration.

In summary, none of the chronic exercise protocols was able to change basal autophagy flux in the liver and heart tissues. However, the moderate/regular exercise models (i.e., endurance, resistance, and concurrent) exhibited normal or enhanced morphological/functionality adaptations with reduced autophagy machinery in these tissues. The OT group presented a higher basal expression of genes responsible for codifying proteins that act as receptors to induce autophagy/mitophagy (*Sqstm1* and *Bnip3*) with negative morphological and physical performance adaptations. In skeletal muscle, the END and OT models reduced the basal autophagy flux in vivo, and the RES model increased this measure. The mechanisms by which different chronic exercise protocols increase or decrease basal autophagy flux in vivo in skeletal muscle must be addressed in future investigations. Additionally, further studies should evaluate the autophagy flux in vivo of trained animals in response to a specific acute exercise session. This measure will clarify how the autophagic adaptations acquired through a long training period will respond to routine stress. Figure 9 summarizes the main findings of the present investigation.

## Supplementary Materials

Supplementary Materials can be found at https://www.mdpi.com/1422-0067/21/22/8416/s1.

## Abbreviations

CT	Control group (sedentary)
END	Endurance; submitted to the chronic endurance exercise protocol
RES	Resistance; submitted to the chronic resistance exercise protocol
CONC	Concurrent; submitted to the chronic concurrent exercise protocol
OT	Overtraining; submitted to the chronic exhaustive exercise protocol
HE	hematoxylin-eosin
PAS	periodic acid Schiff
