# Supplementary material for: Moderate, but Not Excessive, Training Attenuates Autophagy Machinery in Metabolic Tissues
Source: Int J Mol Sci. 2020 Nov 10;21(22):8416. doi: 10.3390/ijms21228416 (PMC7697344; doi:10.3390/ijms21228416)
Supplement: Supplementary file 1 [file ijms-21-08416-s001.pdf]

Supplementary files

Table S1. M and B-mode echocardiogram parameters for the experimental groups at weeks 0, 4, and 8.

**Variables**	**CT group**			**END group**			**RES group**			**CONC group**			**OT group**		
**M-Mode**	**W0**	**W4**	**W8**	**W0**	**W4**	**W8**	**W0**	**W4**	**W8**	**W0**	**W4**	**W8**	**W0**	**W4**	**W8**
IVS;d (mm)	0.7±0.1	0.8±0.1	0.9±0.1	0.8±0.1	0.7±0.1	0.8±0.1	0.7±0.1	0.8±0.1	0.8±0.1	0.8±0.1	0.9±0.1	0.8±0.0	0.8±0.0	0.8±0.0	0.8±0.1
IVS;s (mm)	1.1±0.2	1.1±0.1	1.2±0.1	1.1±0.2	1.0±0.1	1.2±0.1	1.0±0.1	1.2±0.1	1.1±0.2	1.1±0.2	1.2±0.1	1.1±0.1	1.2±0.1	1.1±0.1	1.2±0.1
LVID;d (mm)	3.9±0.3	4.1±0.2	3.8±0.3	4.0±0.3	4.1±0.2	4.2±0.2	3.7±0.2	3.9±0.1	4.0±0.2	3.9±0.2	3.9±0.3	4.1±0.2	4.0±0.2	4.2±0.2	4.2±0.1
LVID; s (mm)	2.9±0.4	3.3±0.3	3.0±0.4	3.1±0.3	3.3±0.2	3.2±0.2	2.9±0.2	2.9±0.2	3.1±0.3	3.1±0.1	3.0±0.4	3.3±0.3	3.1±0.2	3.5±0.3	3.2±0.2
LVPW; d (mm)	0.6±0.1	0.7±0.1	0.8±0.1	0.7±0.1	0.7±0.1	0.7±0.1	0.6±0.1	0.7±0.1	0.7±0.1	0.6±0.1	0.7±0.0	0.6±0.1	0.7±0.1	0.8±0.1	0.6±0.1
LVPW; s (mm)	0.9±0.1	0.9±0.2	1.0±0.2	0.9±0.2	0.9±0.1	1.0±0.2	0.7±0.1	0.9±0.2	0.9±0.2	0.8±0.2	0.8±0.1	0.7±0.2	0.9±0.2	0.9±0.2	0.8±0.1
EF (%)	50.0±7.4	39.0±9.6	45.9±9.1	45.0±5.9	40.5±9.4	48.3±6.3	46.4±7.6	50.9±7.6	44.3±8.1	44.2±4.8	46.6±10.9	40.7±6.3	45.9±9.0	37.7±7.4	47.3±2.6
FS (%)	25.1±4.4	18.8±5.4	22.6±5.4	22.1±3.4	19.7±5.2	24.2±3.8	22.8±4.4	25.7±4.9	21.7±4.6	21.5±2.8	23.2±6.7	19.7±3.5	22.8±5.3	18.1±4.1	23.5±1.5
LV mass (mg)	73.5±14.4	113.3±12.7	94.9±4.2	85.4±13.3	86.4±9.8	99.2±22.1	61.5±11.2	85.4±13.9*	85.3±13.3*	76.3±18.2	86.7±13.0	76.4±6.9	86.6±15.8	100.1±11.6	87.8±12.2
LV vol; d (uL)	67.5±13.4	74.4±6.6	64.3±11.7	71.5±13.7	75.5±10.7	79.8±9.3	59.1±7.6	67.9±4.0	68.5±6.8	66.6±7.1	67.6±12.2	74.8±8.1	71.5±7.2	80.7±10.6	79.1±5.8
LV vol; s (uL)	34.6±12.2	45.7±10.0	35.5±10.3	39.5±9.8	44.7±7.6	41.0±4.7	31.6±5.9	33.2±4.4	38.3±8.4	37.1±3.5	36.8±12.0	44.6±8.3	38.6±7.0	50.5±10.3	41.8±4.8
HR (bpm)	446.8±18.4	407.2±41.5	398.4±65.4	388.0±50.1	382.4±33.0	443.7±51.1	402.9±50.9	330.9±20.3	376.7±33.0	401.6±42.8	371.8±36.7	395.2±35.7	420.2±32.2	421.9±39.1	412.3±66.0
CO (mL/min)	14.2±0.5	13.4±1.2	11.1±1.8	10.9±2.3	11.8±2.9	16.3±5.9	11.9±1.7	11.1±1.3	10.7±1.7	12.0±2.7	12.8±2.1	11.4±1.5	15.1±3.0	15.2±3.3	15.9±2.7
**B-Mode**	**W0**	**W4**	**W8**	**W0**	**W4**	**W8**	**W0**	**W4**	**W8**	**W0**	**W4**	**W8**	**W0**	**W4**	**W8**
Area; d (mm2)	22.5±1.8	24.7±1.0	23.0±2.2	22.4±2.4	23.8±0.7	24.8±2.7	21.1±1.1	23.0±1.9	22.9±1.8	21.6±2.2	24.1±1.2	24.2±1.9	23.6±1.9	26.0±1.2	25.2±1.9
Area; s (mm2)	13.9±2.6	16.3±1.4	15.5±2.5	14.4±2.0	16.4±1.6	16.0±2.3	13.0±0.7	14.1±1.0	15.7±1.1*#	13.5±1.2	15.1±1.5	17.0±1.7	14.0±1.9	17.4±1.3	15.6±1.7
CO (mL/min)	14.3±0.5	13.9±0.7	11.5±2.0	15.8±13.1	11.4±2.6	16.1±5.9	12.1±1.6	11.6±1.2	10.8±1.7	12.3±3.0	13.3±2.3	11.6±1.7	14.7±2.6	15.1±3.9	15.5±3.1
EF (%)	55.0±6.5	49.5±4.8	48.5±9.1	48.6±8.1	46.3±8.6	51.1±9.4	56.3±0.8	55.8±2.3	47.8±5.4	54.9±3.8	54.0±6.2	45.1±3.3*#	57.7±6.1	47.9±6.8	54.7±3.6
FS (%)	13.2±5.6	13.6±2.2	11.0±3.1	17.4±11.7	11.2±4.8	14.8±6.2	13.7±1.9	15.8±3.4	10.1±1.8*	15.3±1.7	14.2±4.5	11.2±1.5	16.2±4.9	13.6±4.9	15.7±3.3
SV (uL)	31.9±1.0	33.0±3.0	28.3±5.5	28.5±7.5	30.6±5.5	35.9±9.5	29.5±2.6	33.7±3.8	28.7±6.3	29.9±4.9	34.4±3.7	28.8±2.8	35.9±5.9	35.8±6.2	38.7±4.6
V;d (uL)	58.8±7.6	66.8±5.4	59.1±10.0	58.4±8.4	66.3±2.4	70.1±11.4	52.4±4.4	60.4±7.2	59.5±8.1	54.4±7.7	63.8±4.7	64.2±7.8	62.3±8.9	74.6±4.5	70.8±8.1
V;s (uL)	26.9±7.3	33.9±5.2	30.8±8.4	29.8±5.6	35.7±6.2	34.2±7.4	22.9±1.9	26.7±3.9	30.8±3.4*#	24.5±3.8	29.5±5.1	35.4±5.8	26.4±5.4	38.7±4.7	32.1±5.1

W0–Week 0; W4–Week 4; W8–Week 8; IVS; d–Interventricular septum thickness at end-diastole; IVS; s–Interventricular septum thickness at end-systole; LVID; d–Left ventricular internal dimension at end-diastole; LVID; s–Left ventricular internal dimension at end-systole; LVPW; d–Left ventricular posterior wall thickness at end-diastole; LVPW; s–Left ventricular posterior wall thickness at end-systole; EF–Ejection fraction; FS–Fractional shortening; LV–Left ventricle; LV vol; d–Left ventricle volume at end-diastole; LV vol; s–Left ventricle volume at end-systole; HR–Heart rate; CO–Cardiac output; SV–Stroke volume; V; d–Volume at end-diastole; V; s–Volume at end-systole. Data correspond to mean ± SEM of 5 mice/group. *$p<0.05$ vs. week 0; #$p<0.05$ vs. week 4. CT (Sedentary; control group); END (Endurance; submitted to the chronic endurance exercise protocol); RES (Resistance; submitted to the chronic resistance exercise protocol); CONC (Concurrent; submitted to the chronic concurrent exercise protocol); OT (Overtraining; submitted to the chronic exhaustive exercise protocol).

Table S2. Primers used to RT-qPCR.

Gene	Forward (5′-3′)	Reverse (5′-3′)
Prkaa1	CCAGGTCATCAGTACACCATCT	TTTCCTTTTCGTCCAACCTTCC
Mtor	CCACGTGGTTAGCCAGACT	TAGCGGATATCAGGGTCAGGA
Ulk1	AACATCCGAGTCAAGATTGCTG	ATAATGACCTCAGGAGCCATGT
Atg5	GCTTTTGCCAAGAGTCAGCTAT	AACCAATTGGATAATGCCATTTCAG
Becn1	AGGAACTCACAGCTCCATTACT	CTCTCCTGAGTTAGCCTCTTCC
Bnip3	CAGCATGAGAAACACAAGCG	TCCAATGTACCCCAAGCC
Map1lc3b	AGATAATCAGACGGCGCTTG	TCGTACACTTCGGAGATGGG
Sqstm1	ACAGCCAGAGGAACAGATGG	GTAGAGACTGGAGTTCACCTGTA
Gapdh	AAGAGGGATGCTGCCCTTAC	CGGGACGAGGAAACACTCTC

Prkaa1: Protein kinase, AMP-activated, alpha 1 catalytic subunit; *Mtor*: Mechanistic target of rapamycin kinase; *Ulk1*: Unc-51 like kinase 1; *Atg5*: Autophagy-related 5; *Becn1*: Beclin1; *Bnip3*: BCL2 interacting protein 3; *Map1lc3b*: Microtubule-associated protein 1 light chain 3 beta; *Sqstm1*: Sequestosome 1/p62; *Gapdh*: Glyceraldehyde-3-phosphate dehydrogenase.
